# SNP-PCR genotyping links alterations in the GABAA receptor (GABRG3: rs208129) and RELN (rs73670) genes to autism spectrum disorder among peadiatric Iraqi Arabs

**DOI:** 10.1007/s11033-022-07388-z

**Published:** 2022-04-11

**Authors:** Zainab A. Ali, Akeel A. Yasseen, Katherine A. McAllister, Arafat Al-Dujailli, Ahmed J. Al-Karaqully, Alaa S. Jumaah

**Affiliations:** 1 Department of Pathology and Forensic Medicine, Faculty of Medicine, University of Kerbala, Kerbala Governorate, Iraq; 2 Department of Pathology and Forensic Medicine, Faculty of Medicine, University of Kufa, P.O. Box 21, Kufa, Iraq; 3 School of Biomedical Sciences, Ulster University, Belfast, Northern Ireland, UK; 4 Psychiatric Department, AL Hussain Teaching Hospital, Kerbala Governorate, Iraq; 5 Department of Pathology and Forensic Medicine, Faculty of Medicine, University of Kufa, P.O. Box 21, Kufa, Iraq

**Keywords:** Pediatric ASD, SNP-biomarkers, RELN, GABRG3

## Abstract

**Introduction:**

Autism spectrum disorder (ASD) is an increasing concern among the Iraqi Arab population. The genetic alterations that cause ASD are likely to converge at the synapse. This study investigated polymorphisms in the GABA_A_ receptor subunit (GABRG3) and the RELN gene as putative biomarkers of ASD in a pediatric population in Iraq.

**Methods:**

The case control study included 60 patients with a clinical diagnosis of ASD (mild, moderate, or severe) according to DSM-IV criteria and matched healthy controls (n = 60). Blood samples were collected for DNA genotyping of SNPs rs736707 and rs208129 for RELN and GABRG3 using allele specific PCR. Assessment of genotype and allele distributions in patient groups used odd ratios (OR) with 95% confidence intervals and the Chi-square test. All statistical analysis was performed used SPSS software.

**Result:**

The patient cohort was highly consanguineous, with increased ratio (p > 0.05) of males to females (3:1) in both ASD (mean age, 6.66 ± 3.05) and controls (mean age, 5.76 ± 2.3). Both GABRG3 rs208129 genotypes TT (OR 4.33, p = 0.0015) and TA (OR 0.259, P = 0.008), and the T and A alleles were significantly associated with ASD. The RELN rs736707 TC genotype (OR 2.626, P = 0.034) was the only significant association with ASD.

**Conclusion:**

GABRG3 SNP rs208129 is a leading biomarker to predict genetic vulnerability to ASD in Iraqi Arabs. Expanded SNP panels and increased sample sizes are required for future GABRG3 studies, and to reach a consensus on RELN utility. Future ASD screening programs in Iraq should include genetic metrics in addition to clinical phenotype assessments.

**Supplementary Information:**

The online version contains supplementary material available at 10.1007/s11033-022-07388-z.

## Introduction

Autism spectrum disorder (ASD) is a neurodevelopmental condition that causes impaired verbal and nonverbal communication, reciprocal social interactions, and stereotypic behaviors, interests, and activities in affected children [1, 2]. The median prevalence of ASD is 62 out of 10,000 individuals, with a reported four to one male bias [3]. The early diagnosis of ASD and intervention treatments can improve social and cognitive abilities of patients in later life [4]. This reduces burden and costs to society in the long run. Currently there is a worldwide bottleneck in the age of diagnosis of children with ASD [5]—however this could be addressed by identification of genetic biomarkers for the disorder. Particularly in the Iraqi nation, there is a paucity of genetic and molecular studies of ASD to provide adequate diagnosis in the clinic [6].

Emerging evidence suggests that genetic variants especially single nucleotide polymorphisms (SNP) are associated with ASD. While no single gene can account for the heterogeneity of symptoms occurring in patients with ASD; identical twin studies show that autism heritability estimates are substantial (64–91%) [7]. Owing to these high heritability estimates, a major focus of autism research is to identify the candidate genes involved [8]. Early brain development genes, including synapse formation and stabilization, and neurotransmission in neutral circuits are consistently associated with autism [9, 10]. Therefore ASD is believed to be a ‘disease of the synapse’.

The Autism Sequencing Consortium identified RELN with a 95% probability of being a gene whose anomalies directly contribute to autism [11]. Genome scans indicate a linkage of autism to the chromosome 7q21–q36 [12, 13]. The Reelin gene (RELN) is located on chromosome 7q22 and codes for a large extracellular glycoprotein with serine protease activity in humans. Reelin is found in the brain, blood, spinal cord, and other organs and tissues throughout the body. Reelin exerts several important functions in the brain including the regulation of neuronal migration, dendritic growth and branching, dendritic spine formation, synaptogenesis, and synaptic plasticity [14]. Postmortem studies show reduced reelin protein levels in the brain and plasma of autistic individuals [15]. The use of RELN SNPs to determine susceptibility to ASD is still contradictory. For instance, a 2017 meta-analysis indicated that RELN rs736707 contributed significantly to ASD risk in Asians compared to Caucasians [16]. Yet a recent study among children and adolescents with ASD in the Tianjin, China population suggests no association [17, 18].

The development of ASD is also linked to decreased inhibitory neurotransmission signals (increased excitatory) in the brain synapses. Gamma-aminobutyric (GABA) is the most common inhibitory neurotransmitter in the central nervous system. The recognition of GABA by the GABA_A_ receptor causes rapid post synaptic inhibition in the brain. Reduced GABA_A_ receptors in the superior frontal cortex of ASD patients are a source of ASD symptoms [19, 20]. There are a cluster of GABA_A_ receptor subunit genes located in chromosome 15q (GABRB3, GABRA5, and GABRG3) that are implicated in the etiology of ASD [21]. The GABA_A_ receptor subunit gene polymorphisms (GABRB3 rs2081648, GABRA5 rs35586628, and GABRG3 rs208129) predict symptom-based and developmental deficits in Chinese Han children and adolescents with ASD [22].

To reach a consensus on usefulness of ASD susceptibility biomarkers for the Iraqi Arab population, this study selected two key polymorphisms for investigation—GABRG3, (rs208129) and RELN (rs736707). Here we report the findings and significance of the genotype and allele distributions identified in patients with ASD according to severity subtypes.

## Materials and methods

### Study population

A total of 60 ASD patients and 60 healthy controls were recruited to the present investigation (N = 120). All patients were subjected to thorough psychological assessments by two consultant psychiatrists independently. The ASD subjects fulfilled the DSM-IV criteria for diagnosis. The ASD patients were re-grouped according to severity by psychiatrists. The 60 age and sex matched normal children were selected from the local teaching hospital as a control group. The control group was also subjected to psychological examination to rule out any neurological disorders or learning difficulties. The families of both groups were contacted and informed about the aim of the project. Those who agreed and allowed their children to participate were given a formal written consent to sign and questionnaire to complete. The project was approved by the Institutional Review Board of the University of Kufa, Faculty of Medicine, in accordance with the 1964 Helsinki declaration and its later amendments. The study was conducted at the Department of Pathology and Forensic Medicine, Faculty of Medicine, University of Kufa.

### Blood sample collection and genomic DNA experiments

Peripheral blood volumes (5.0 mL) were withdrawn from both healthy and ASD patients in parallel and inoculated into EDTA tubes. The blood tubes were transferred on ice to the Department of Pathology for molecular analysis. Genomic DNA was isolated from blood samples using the Bosphore® Genomic DNA Extraction Spin Kit according to the manufacturer instructions. The isolated genomic DNA was analyzed using the Nano-drop spectrophotometer. DNA was measured using a wavelength of 260 nm, and concentrations expressed in terms of ng/µL.

Agarose gel electrophoresis was performed as mentioned previously (Sambrook and Fritsch 1989). Briefly, 1.0 g agarose was added to 100 mL of TAE buffer (1X). The mixture was heated to completely melt the agarose mixture. Then, ethidium bromide, at a final concentration of 0.5 µg/mL, was added to the melted agarose. The melted agarose was poured into the casting tray of the submarine gel electrophoresis unit (Cleaver Scientific Co., UK). After gel solidification, the casting tray was immersed in the submarine tank. The TAE buffer (1X) was added until submerging the gel under the surface of the buffer completely. Each DNA sample was mixed with 1X gel loading dye prior to loading into the gel. The electrophoresis process was conducted at 5–8 Voltage/cm between the anode and the cathode for 45 min. After termination of electrophoresis, the agarose gel was visualized under the ultra-violet (UV) light using a UV-transilluminator (Cleaver Scientific Co., UK).

### Allele specific PCR primer design and synthesis

All allele specific PCR primers used in this study were designed manually. Briefly, the questionable SNPs were retrieved from dbSNP (a database containing human single nucleotide polymorphism variations, microsatellites, small scale-insertions and deletions insertion located at the server https://www.ncbi.nlm.nih.gov/snp/). Mostly, a nucleotide sequence of 1000 bp or 500 bp containing the questionable SNP was retrieved. Then, the allele specific primers were designed manually and were computationally checked regarding 3′ complementarity, 3′self -complementarity, GC content, and melting temperature using the primer-blast online program, localized at the server (https://www.ncbi.nlm.nih.gov/tools/primer-blast/) from NCBI (National Center for Biotechnology and Information). All designed primers in this study were synthesized in Integrated DNA Biotechnology (IDT Co., Canada).

The SNPs of accession numbers rs736707 and rs208129 of the Reelin and GABRG3 genes were genotyped using the study participant DNA and the allele specific PCR technique. Each DNA sample was processed through two alleles specific PCR reactions. Each allele specific PCR reaction was directed with a primer set: forward primer carrying the allele SNP base at 3′ prime end and a reverse primer. Results were read according to the guidelines. Supplementary Tables 1 and 2 respectively show the allele specific primer sequences and PCR conditions for the GABRG3 and RELN SNPs.

### Statistical analysis

The Statistical Package of Social Sciences version 27 (SPSS Inc.; Chicago, IL, USA) computer program was used for results analysis. For each variable the values are presented as mean ± SD. Student t test was used to determine the statistical difference between two groups. One-Way ANOVA was performed to evaluate the differences among multiple groups. For statistical comparison between different groups, the statistical significance was defined as p ≤ 0.05, while a p-value of > 0.05 was no significant. A p-value of < 0.001 was considered highly significant. In the studied groups, the representativeness of alleles and genotypes was estimated by the Hardy–Weinberg equilibrium (HWE) by comparing the observed and expected frequencies of genetic variants. Chi-square test was applied to assess genotype and allele frequencies between patients and controls. The genotype and allele distributions were determined in each group, and odds ratios (OR) with 95% confidence intervals (95% CI) were calculated. A p-value of < 0.05 was considered statistically significant at a confidence interval (CI) of 95%.

## Result

### Patient characteristics

**Fig. 1:**
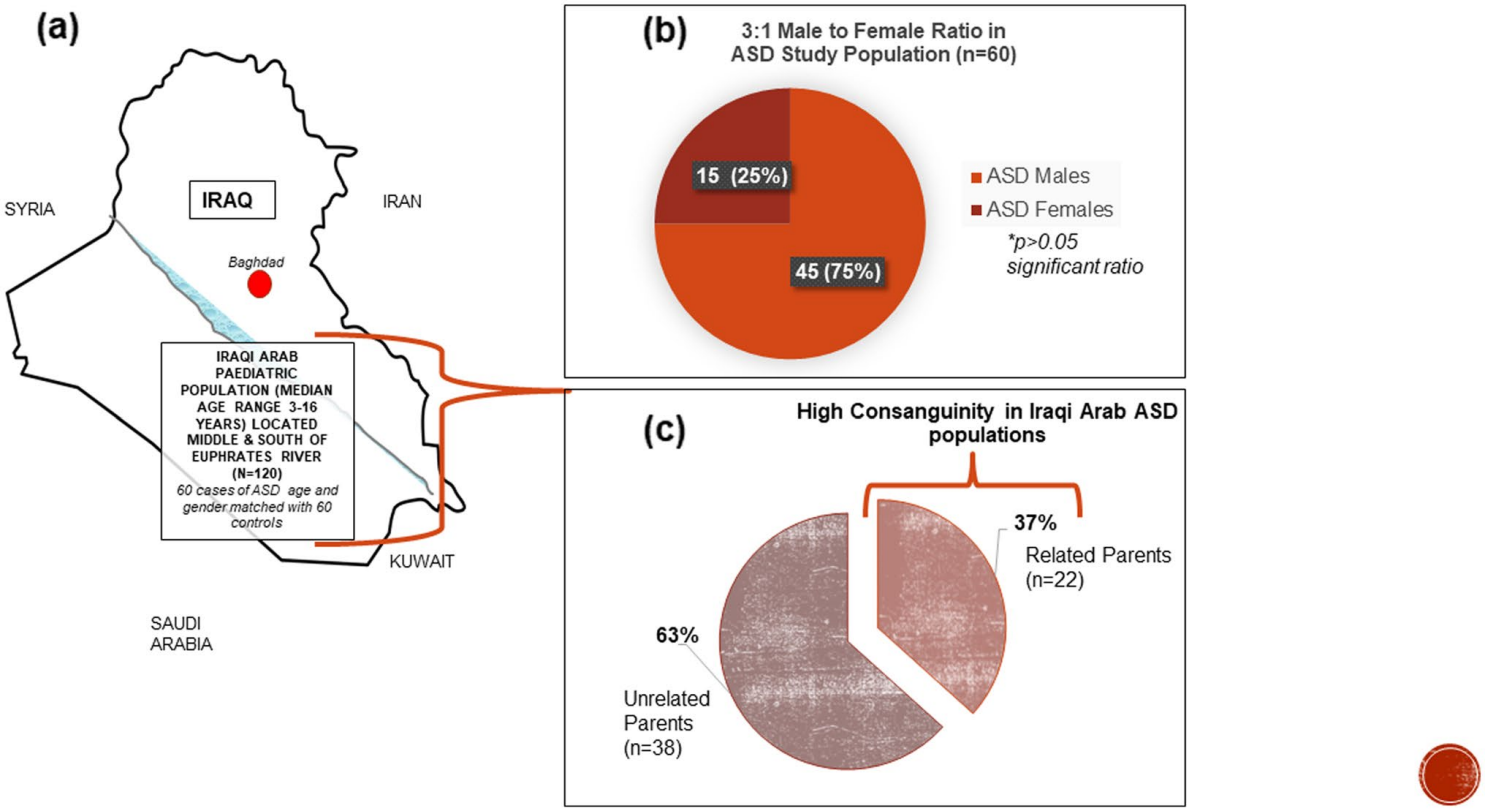
**a** Iraqi Arab population was located geographically Middle and South of the Euphrates River. **b** 3:1 Male to Female Ratio in ASD Study Population. **c** High Consanguinity in Iraqi Arab ASD populations

The mean age of patients with ASD was 6.66 ± 3.05 (median range 3–16) and that for controls was 5.76 ± 2.3 (median range 3–16) years. There was a significantly higher (p > 0.05) ratio of males to females (3:1) for both the ASD (n = 60) and matched healthy control populations (n = 60). The rate of consanguinity was high at 36.66% and 40% in both the ASD and control populations respectively. Figure 1a–c shows the location of the entire study population (n = 120) of Iraqi Arabs in the Middle and South Euphrates region; in addition to the gender and consanguinity distributions (illustrated for the ASD cases only).

Table 1 shows the demographic characteristics of the patients with ASD according to the DSM-IV severity classification. The majority of ASD cases were characterized as being mild (n = 39) in the severity of autism symptoms according to the American Psychiatric Association’s Manual of Psychiatric Diseases, 4th edition (DSM-IV). Some of the mild cases (n = 13) were the children of consanguineous marriages (35.89%; 95% CI 0.41180–2.2381, OR 0.967). A total of 13 patients were classified as moderate severity, of which four were the product of consanguineous marriages (30.76%; 95% CI 0.2114–2.7876, OR 0.767). The odd ratio for consanguinity was highest in the patients with severe ASD classification (n = 8, 50%; 95% CI 0.3924–7.6035, OR 1.727).

**Table 1:** Demographic characteristics of the patients with ASD according to DSM-IV severity classification

ASD classification	No	Mean Age ± SD (Years)	Consanguinity	Unrelated parents	OR	P value	95% CI
ASD (unclassified)	60	6.66 ± 3.05	22 (36.66%)	38 (63.33%)	1	1	0.4759–2.1014
DSM-IV: mild	39	5.98 ± 2.53	14 (35.89%)	25 (64.1%)	0.967	0.938	0.4180–2.2381
DSM-IV: moderate	13	7.230 ± 1.92	4 (30.76%)	9 (69.23%)	0.767	0.687	0.2114–2.7876
DSM-IV: severe	8	9.71 ± 4.82	4 (50%)	4 (50%)	1.7273	0.469	0.3924–7.6035

*ASD* Autism spectrum disorder, *DSM-IV* diagnostic and statistical manual of mental disorders, (American Psychiatric Association) fourth edition

### Genotype and allele distribution of GABRG3 gene SNP rs208129

**Fig. 2:**
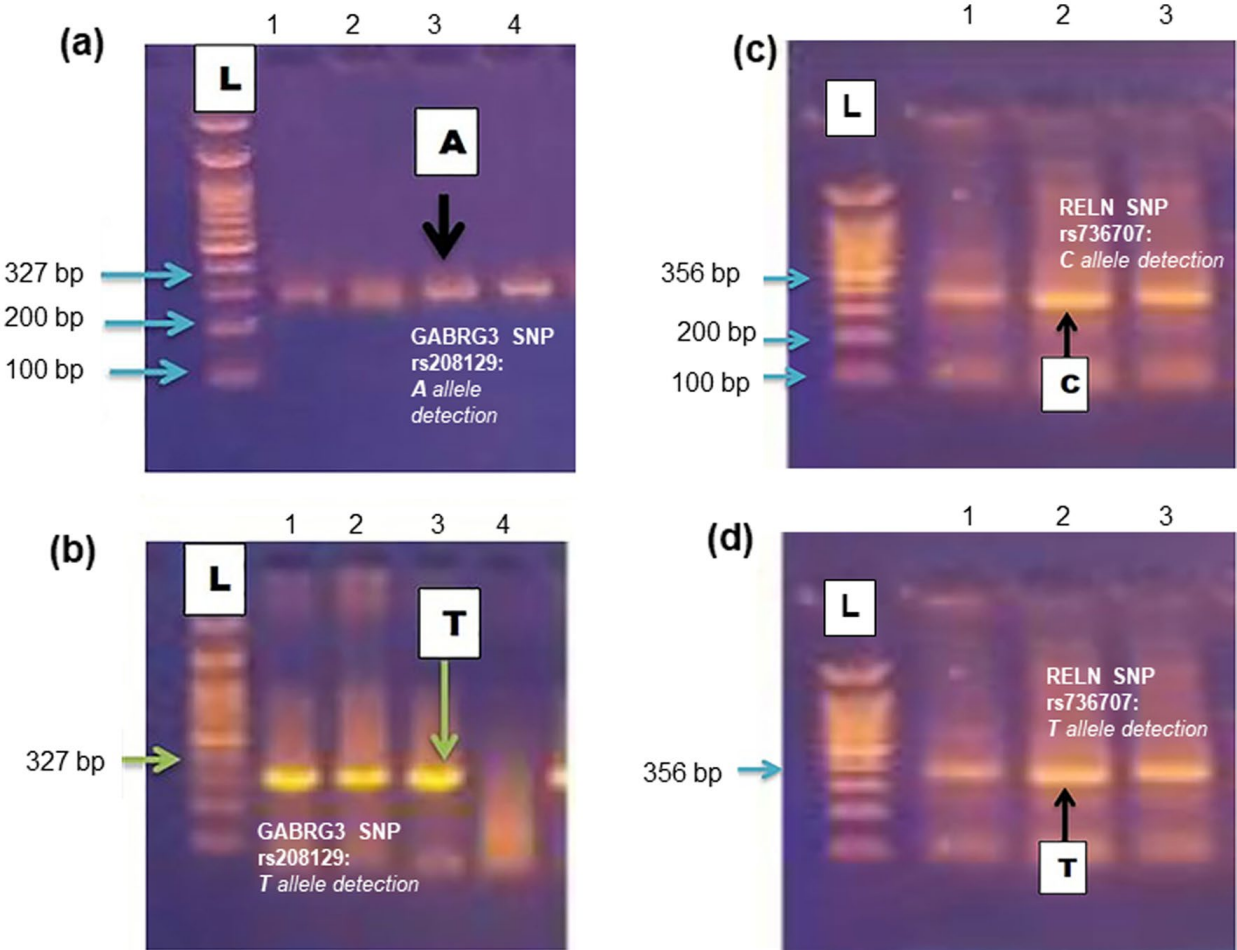
**a** and **b** shows A and T allele detection by gel electrophoresis for GABRG3, **c** and **d** shows C and T allele detection for RELN

Table 2 reports the allele and genotype distributions of the GABRG3 SNP rs208129 (T/A) in the study population. Figure 2a–b shows the results of the A and T allele detection by gel electrophoresis. Most patients with ASD showed higher frequency distribution (86.66% versus 60.0%) and significant association with the TT genotype in comparison to healthy controls (OR 4.33; 95% CI 1.7512; P = 0.005 (HS) comparisons to healthy controls. The TA genotype occurred at a significantly reduced frequency in patients with ASD (n = 6, 10%) versus controls (n = 18, 30% (OR 0.259; 95% CI 0.0946–7.1050; P = 0.008). Finally, the AA genotype frequency was found in only 2 cases of ASD (3.33% versus 10%) (OR 0.3103; 95% CI 0.0600–1.6042; P = 0.1627).

**Table 2:** Genotype (TT, TA, AA) and allele (T, A) distributions of GABAG3 rs208129

Genotype/allele	Patients (N = 60)		Control (N = 60)		P value	OR	95% CI
	No	%	No	%			
*Genotype*							
TT	52	86.67	36	60	0.0015*	4.33	1.7512 to 10.722
TA	6	10	18	30	0.008*	0.259	0.0946 to 7.1050
AA	2	3.33	6	10	0.1627	0.3103	0.0600 to 1.6042
*Alleles*							
T	110	91.67	90	75	0.0009**	3.666	1.7011 to 7.9033
A	10	8.33	30	25	0.0009**	0.272	0.1265 to 0.5878

No significant association (P > 0.05); *Significant association (P < 0.05); **Highly significant association (P < 0.001)

The frequency of T allele carriers (91.66%, n = 110 versus 75%, n = 90) had a highly significant association with the ASD compared to the control population (OR 3.66; 95% CI 1.7011–7.9033; P = 0.0009). Whereas the A allele distribution frequency was significantly reduced (8.33%, n = 10) in the ASD population (OR 0.272; 95% CI 0.1265–0.5878; P = 0.0009).

There were no significant differences in the genotype and allele distribution of SNP rs208129 in GABRG3 gene according to the gender (Supplementary Table 3).

### GABAG3 rs208129 polymorphism distribution in the ASD severity categories

The distribution of the genotypes and alleles for SNP rs208129 in GABRG3 gene according to the severity of the symptoms in ASD are displayed in Table 3. The TT genotype occurred in the majority of mild (n = 35, 89.74%), moderate (n = 9, 69.23%) and all cases of severe ASD (n = 8, 100%). There was no significant association in the frequencies of the TT genotype among mild, moderate, and severe cases of SD (P = 0.207) nor TA genotype (P = 0.519). The AA genotype frequency occurred in two patients with moderate ASD (P = 0.024). The Chi-square test found significant T and A allele distributions among mild, moderate, and severe cases of ASD (χ^2^ = 9.90, df = 2, p = 0.007).

**Table 3:** GABAG3 rs208129 polymorphism according to ASD severity categories

Genotype/allele	Mild N = 39		moderate N = 13		Severe N = 8		Df	*X*^*2*^	P-value
	No	%	No	%	No	%			
*Genotype*									
TT	35	89.74	9	69.23	8	100	2	3.15	0.207
TA	4	10.25	2	15.38	0	0	2	0.131	0.519
AA	0	0	2	15.38	0	0	2	7.48	0.024*
*Alleles*									
T	74	94.87	20	76.92	16	100	2	9.90	0.007*
A	4	5.12	6	23.07	0	0	2	9.90	0.007*

No significant association (P > 0.05); *Significant association (P < 0.05); **Highly significant association (P < 0.001)

### Genotype (TT/TC/CC) and allele distribution of RELN gene SNP rs736707

Table 4 shows the genotype and allele distribution of the RELN gene rs736707 in the study population. Figure 2c and d shows the results of the A and T allele detection by gel electrophoresis. There was no significant association in the distribution of the TT genotype in the ASD population autistic relative to healthy controls (OR 0.619; 95% CI 0.2812–1.3630, OR 0.619 P = 0.316). On the other hand, the TC genotype had a significantly stronger association in ASD cases (n = 19, 30%) when compared to controls (OR 2.626; 95% CI 1.0748–6.4161; P = 0.034). Finally, the CC genotype distribution had a weak association with ASD among two patients (3.33%) out of the total cohort (OR 0.310; 95% CI 0.0600–1.6042; P = 0.162).

**Table 4:** Distribution of the genotypes (TT, TC, CC) and alleles (T, C) of RELN gene rs736707

Genotype/allele	Patients N = 60		Control N = 60		P value	OR	95% CI
	No	%	No	%			
*Genotype*							
TT	39	66.67	45	75	0.316	0.619	0.2812–1.3630
TC	19	30	9	15	0.034*	2.626	1.0748 to 6.4161
CC	2	3.33	6	10	0.162	0.310	0.0600 to 1.6042
*Alleles*							
T	98	81.67	99	82.5	0.866	0.944	0.4884 to 1.8281
C	22	18.33	21	17.5	0.866	1.583	0.5470 to 2.0475

No significant association (P > 0.05); *Significant association (P < 0.05)

As for the allele distribution, T and A carriers occurred at similar frequencies in both the ASD and control population (P > 0.05).

There were no significant differences in the genotype and allele distribution RELN gene rs736707 according to gender (Supplementary Table 1).

### RELN rs736707 polymorphism distribution in the ASD severity categories

The distribution of the genotypes and alleles for SNP RELN rs736707 according to the severity of the symptoms in ASD are displayed in Table 5. There were no significant differences in the genotypes (TT, TC, CC) or alleles (T, C) among the ASD subtypes.

**Table 5:** RELN rs736707 polymorphism according to ASD severity categories

Genotype/allele	Mild N = 39		Moderate N = 13		Severe N = 8		Df	*X*^2^	P-value
	No	%	No	%	No	%			
*Genotype*									
TT	25	64.10	9	69.24	5	62.5	2	0.138	0.933
TC	12	30.77	4	30.76	3	37.5	2	0.145	0.930
CC	2	5.13	0	0	0	0	2	1.11	0.573
*Alleles*									
T	62	79.48	22	84.62	13	81.25	2	0.333	0.847
C	16	20.52	4	15.38	3	18.75	2	0.333	0.847

## Discussion

Currently the diagnosis of ASD is based on the phenotypic presentation of symptoms in patients. Since the disorder is genetically heterogenous, patients would benefit greatly from diagnostic genetic testing. Therefore future treatment approaches must involve patient stratification according to genetics for improved treatment (personalized medicine). The world health organization has recognized the need to strengthen the abilities of countries across the globe to support the health and wellbeing of individuals with autism [23]. In addition to being deprived of adequate genetic diagnosis and healthcare during the critical early years, individuals with ASD can be the subject of stigma and discrimination that prevents them from participating fully in their communities in later life. Autism can also be affected by living in a volatile environment—which is the case in many Middle East countries such as Iraq.

There is presently a paucity of genetic studies of ASD in the Iraqi Arab ethnic population to provide adequate diagnosis, support, and issue guidance for the future [6]. The early genetic diagnosis of ASD in children is a key strategy for introducing treatment and/or intervention programs to improve outlook in adulthood. The genetic alterations that cause ASD are likely to converge at the synapse and may involve the GABA_A_ receptor and Reelin signaling. Therefore candidate SNPs for identifying ASD in the RELN and GABRG3 genes were investigated in a young population of Iraqi Arabs located in the Middle and Southern Euphrates region. This study paves the way for introducing population-relevant genetic biomarkers for the early detection and better management of ASD throughout Iraq.

The study found a high rate of consanguinity (36.66% and 40%) in both the ASD and control populations of Iraqi Arabs. The risk of producing offspring with ASD increases from blood-related marriages [24]. While consanguinity occurs in an estimated 10.4% of the global population [25], Arabs have the highest rates, approaching 60% in Iraq [26]. Therefore the evaluation and diagnosis of ASD in Arab communities should entail screening for consanguinity, and preconception genetic counseling offered to families with a history of ASD.

Autism also has a sex bias with up to five times higher frequency amongst boys compared to girls [3]. Our study had a 3:1 male to female ratio in the ASD population. The DSM-5 states that “autism spectrum disorder is diagnosed four times more often in males than in females.” [27]. Although the 4:1 gender ratio is widely cited, most recent research suggest that it is closer to 3:1 [28]. Therefore our study population is a reflective of the gender ratio in ASD and provides a useful model to explore specific genetic associations. The results of this study found no significant differences in the genotype and allele distribution of both RELN and GABRG3 SNPs between males and females with ASD. Therefore the development of ASD occurring from genetic alterations in RELN and GABRG3 is independent of gender in the Iraqi Arab population.

However a key finding of the present investigation was the significant difference in the distribution of the GABRG3 genotypes TT (OR 4.33, P = 0.005) and TA (OR 0.259, P = 0.008) in the ASD cases compared to the healthy controls. This indicates that the TT genotype is more predominant and expressed in the ASD persons while the TA is reduced. The highly significant distribution frequency of T allele (OR 3.66) provided further evidence that T carriers are more predominant in young Iraqi Arab ASD patients than normal. This variation in the TT allele frequency may compromise the GABA_A_ receptor critical function of post synaptic inhibition in the brain. This may affect GABA processing that results in altered behaviors, cognition, and the body's response to stress. A key study of Chinese autistic children showed that the TT and TA genotypes of GABRG3 SNP rs208129 had more severe deficits in ‘imitative behavior’ and ‘activity level’ than children with the AA genotype [22]. The present study result of stratified ASD subtypes showed that all the severe cases of ASD had the TT genotype. There was found to be a significant association of both presence of the T allele and absence of the A allele according to the severity of ASD (p = 0.007). The GABRG3 polymorphism rs208129 can therefore be used as a functional biomarker to predict susceptibility to ASD in young Iraqi Arabs.

Many studies link alterations in the GABA receptor to ASD. A nominal association between the GABRG3 gene and ASD has also been observed in a Caucasian cohort [29] while a Chinese ASD cohort [17, 18] found a positive association with SNP rs7180500. [30] also identified a significant association and gene–gene interaction of GABA receptor subunit genes in autism. A recent Indian study investigated five GABA receptor type A variants in ASD: GABRB3 (rs4906902, rs7171660), GABRG3 (rs208129, rs140679), GABRA5 (rs 140681) [31]. The investigators found that all variants were associated with at least one deficit in ASD-associated phenotypes like ‘relating to people’, ‘imitation’, ‘emotional response’, ‘body use’, ‘taste, smell, touch response’ and ‘activity levels’.

However autism has a polygenic inheritance, thus the effects of some SNPs may be subtle or inconsistent. Variability in SNP genotyping results can also be caused by the diverse genetic heterogeneity in a research study population of ASD. Indeed, the association of the RELN gene SNP rs736707 with ASD development was less significant than GABRG3 in the Iraqi Arab population. However the TC genotype distribution frequency was significantly increased (OR 2.626, p = 0.034) in the RELN gene of ASD subjects. No significant differences were observed between ASD cases and the healthy subjects regarding the T and C allele distribution. There was also no significant difference in RELN rs736707 genotypes and alleles among stratified patients according to mild, moderate, and severe ASD. Therefore at present, use of the reelin polymorphism rs736707 as a functional molecular biomarker to predict susceptibility for ASD in the Iraqi Arab population requires additional research. Several studies have indicated there is no association of rs736707 with ASD [32–34]. However, in South African populations, a significant association of SNP rs736707 but not for SNP rs362691 is observed [35]. Furthermore a study of the eight SNPs of the RELN gene detected a positive association of rs736707 with autism in the Chinese Han Population [36]. A recent study among children and adolescents with ASD in the Tianjin, China population found that although there was no significant association of RELN rs736707 with ASD risk, there was a correlation with the Antecedent, Behavior, Consequence (ABC) sensory subscale and total score [17, 18],

In conclusion, future large-scale population studies of both GABRG3 rs208129 and RELN rs736707 are necessary to confirm the results in the Iraqi population, in addition to screening an expanded panel of GABA_A_ receptor subunit and RELN SNPs. Despite some contradictory studies [37, 38] it appears that GABA_A_ receptor subunit gene variants such as GABRG3 rs208129 provide a reliable metric for assessing ASD and severity subtypes in the Iraqi Arab population. The clinical diagnosis of ASD is based on behavioral observation rather than an objective metrics. However patients with ASD have diverse clinical presentations as the disorder has such variable genetics. In the future, genetic metrics can guide treatment plans for specific social deficit symptoms of the individual patient with ASD. For example, at first glance GABA appears a panacea to address the multiple symptoms of autism spectrum disorder, including agitation and anxiety. However, patients with ASD related to GABA receptor genetic alterations might not benefit from supplementation, as neurobiological defects in post synaptic function may prevent them from effectively utilizing the additional GABA. Conversely, cases of ASD that are unrelated to genetic GABA receptor alterations might benefit from GABA therapies. The successful exploitation of genetic biomarkers for diagnosis, classification, and treatment of ASD will require personalized medicine approaches at the level of the individual patient.

## Supplementary Information

Below is the link to the electronic supplementary material.

Supplementary file1 (DOCX 14 kb)

Supplementary file2 (DOCX 14 kb)

Supplementary file3 (DOCX 16 kb)
