# Supplementary material for: SNP-PCR genotyping links alterations in the GABAA receptor (GABRG3: rs208129) and RELN (rs73670) genes to autism spectrum disorder among peadiatric Iraqi Arabs
Source: Mol Biol Rep. 2022 Apr 11;49(7):6019–28. doi: 10.1007/s11033-022-07388-z (PMC9270290; doi:10.1007/s11033-022-07388-z)
Supplement: Supplementary file 1 — Supplementary file1 (DOCX 14 kb) [file 11033_2022_7388_MOESM1_ESM.docx]

**Supplementary Table S1.** PCR recipe, primer design and PCR conditions for allele specific PCR for GABRG3 rs208129 (A/T)

| **SNP and Primer sequence** | **PCR recipe ^a^** | **PCR conditions ^b^** | **PCR product (bp)** |
| --- | --- | --- | --- |
|  | Genomic DNA: 3.0 µL  Forward-primer: 2.0 µL  Reverse-primer: 2.0 µL  2XPCR master  mix: 12.5 µL  NFW^c^: 5.5 µL  Total volume: 25 µL | 1 cycle:  Initial denaturation: 95 ^o^C, 5 minutes    30 cycles: each cycle  Denaturation: 94 ^o^C, 1 minute  Annealing: 58 ^o^C, 1 minute  Extension: 72 ^o^C, 30 seconds    1cycle:  Final extension: 72 ^o^C, 10 minutes | 327 |
| Allele specific PCR (A allele), 1^st^ PCR reaction |  |  |  |
| Forward-GABRG3- rs208129-allele A  5’- GCATCTTTGGCTTCCATCCT**A** -3’    Reverse-GARG3- rs208129  5’- ATC TTC CCC TGA TTT TTG TGA TAC -3’ |  |  |  |
| GABRG3-Allele specific PCR (T allele), 2^nd^ PCR reaction |  |  | 327 |
| Forward-GABRG3- rs208129-allele T  5’- GCATCTTTGGCTTCCATCCT**T** -3’    Reverse-GARG3- rs208129  5’- ATC TTC CCC TGA TTT TTG TGA TAC -3’ |  |  |  |

a: PCR recipe for both PCR reactions. b: PCR conditions for both PCR reactions. NFW: nuclease free water. Bold base: refers the position of allele SNP.
