# Supplementary material for: SNP-PCR genotyping links alterations in the GABAA receptor (GABRG3: rs208129) and RELN (rs73670) genes to autism spectrum disorder among peadiatric Iraqi Arabs
Source: Mol Biol Rep. 2022 Apr 11;49(7):6019–28. doi: 10.1007/s11033-022-07388-z (PMC9270290; doi:10.1007/s11033-022-07388-z)
Supplement: Supplementary file 2 — Supplementary file2 (DOCX 14 kb) [file 11033_2022_7388_MOESM2_ESM.docx]

Supplementary Table S2. PCR recipe, primer design and PCR conditions for allele specific PCR for RELN rs736707 (C/T)

| **SNP and Primer sequence** | **PCR recipe ^a^** | **PCR conditions ^b^** | **PCR product size (base pair)** |
| --- | --- | --- | --- |
|  | Genomic DNA: 3.0 µL  Forward-primer: 2.0 µL  Reverse-primer: 2.0 µL  2XPCR master  mix: 12.5 µL  NFW: 5.5 µL  Total volume: 25 µL | 1 cycle:  Initial denaturation: 95 ^o^C, 5 minutes    30 cycles: each cycle  Denaturation: 94 ^o^C, 1 minute  Annealing: 60 ^o^C, 1 minute  Extension: 72 ^o^C, 25 seconds    1cycle:  Final extension: 72 ^o^C, 10 minutes | 356 |
| Allele specific PCR (T allele), 1^st^ PCR reaction |  |  |  |
| Forward-RELN- rs736707-allele T  5’- CAGAGGCTGGAGGCAGAA**T** -3’    Reverse- RELN- rs736707  5’- T CAG ATC CCT CAG GCT GGA G -3’ |  |  |  |
| Allele specific PCR (C allele), 2^nd^ PCR reaction |  |  | 356 |
| Forward-RELN- rs736707-allele C  5’- CAGAGGCTGGAGGCAGAA**C** -3’    Reverse- RELN- rs736707  5’- T CAG ATC CCT CAG GCT GGA G -3’ |  |  |  |

a: PCR recipe for both PCR reactions. b: PCR conditions for both PCR reactions. NFW: nuclease free water. Bold base: refers the position of allele SNP. N.B: rs736707 is diallelic (C/T).
