# Supplementary material for: SNP-PCR genotyping links alterations in the GABAA receptor (GABRG3: rs208129) and RELN (rs73670) genes to autism spectrum disorder among peadiatric Iraqi Arabs
Source: Mol Biol Rep. 2022 Apr 11;49(7):6019–28. doi: 10.1007/s11033-022-07388-z (PMC9270290; doi:10.1007/s11033-022-07388-z)
Supplement: Supplementary file 3 — Supplementary file3 (DOCX 16 kb) [file 11033_2022_7388_MOESM3_ESM.docx]

**Supplementary Table 3:** Genotype and allele distribution of GABRG3 SNP rs208129 and RELN SNP rs736707 among ASD group according to the gender

| GABRG3  Genotype / allele | Male  N=45 | | Female  N=15 | | DF | X^2^ | P-value | | |  |  |  |
| --- | --- | --- | --- | --- | --- | --- | --- | --- | --- | --- | --- | --- |
|  | No. | % | No. | % |  |  |  |  |  |  |  |  |
| Genotype: | | | | | | | | | |  |  |  |
| TT | 38 | 84.44 | 14 | 93.33 | 1 | 0.769 | 0.380(NS) | | |  |  |  |
| TA | 5 | 11.11 | 1 | 6.66 | 1 | 0.247 | 0.619(NS) | | |  |  |  |
| AA | 2 | 4.44 | 0 | 0 | 1 | 0.690 | 0.406(NS) | | |  |  |  |
| Alleles | | | | | | |  | | |  |  |  |
| T | 81 | 90 | 29 | 96.66 | 1 | 1.309 | 0.253(NS) | | |  |  |  |
| A | 9 | 10 | 1 | 3.04 | 1 | 1.309 | 0.253(NS) | | |  |  |  |
| RELN  Genotype / allele | Male  N=45 |  | Female  N15 |  | DF | X^2^ | |  |  | P-value | | |
|  | No. | % | No. | % |  |  |  |  |  |  |  |  |
| Genotype | | | | | | | | | |  |  |  |
| TT | 30 | 66.67 | 9 | 60 | 1 | 0.220 | 0.639(NS) | | |  |  |  |
| TC | 13 | 28.89 | 6 | 40 | 1 | 0.642 | 0.423(NS) | | |  |  |  |
| CC | 2 | 4.44 | 0 | 0 | 1 | 0.690 | 0.406(NS) | | |  |  |  |
| Alleles | | | | | | |  | |  |  |  |  |
| T | 73 | 81.12 | 24 | 80 | 1 | 0.018 | 0.893(NS) | | |  |  |  |
| C | 17 | 18.88 | 6 | 20 | 1 | 0.018 | 0.893(NS) | | |  |  |  |

NS: not significant
